# Surprise positive culture rate in the treatment of presumed aseptic long-bone nonunion: a systematic review with meta-analysis of 2397 patients

**DOI:** 10.1007/s00402-023-05103-6

**Published:** 2023-11-25

**Authors:** Robert Kaspar Wagner, Clinton Hugo van Trikt, Caroline E. Visser, Stein J. Janssen, Peter Kloen

**Affiliations:** 1 Department of Orthopedic Surgery and Sports Medicine, Amsterdam UMC Location University of Amsterdam, Meibergdreef 9, 1105 AZ Amsterdam, The Netherlands; 2 Amsterdam Movement Sciences, Musculoskeletal Health, Amsterdam, The Netherlands; 3 Department of Medical Microbiology and Infection Prevention, Amsterdam Institute for Infection and Immunity, Amsterdam UMC Location University of Amsterdam, Amsterdam, The Netherlands

**Keywords:** Nonunion, Infection, Culture, Etiology, Microbiology, Outcome

## Abstract

**Introduction:**

In pre-operatively presumed aseptic nonunions, the definitive diagnosis of infection relies on intraoperative cultures. Our primary objective was to determine (1) the rate of surprise positive intraoperative cultures in presumed aseptic long-bone nonunion (surprise positive culture nonunion), and (2) the rate of surprise positive cultures that represent infection vs. contamination. Secondary objectives were to determine the healing and secondary surgery rates and to identify cultured micro-organisms.

**Materials and Methods:**

We performed a systematic literature search of PubMed, Embase and Cochrane Libraries from 1980 until December 2021. We included studies reporting on ≥ 10 adult patients with a presumed aseptic long-bone nonunion, treated with a single-stage surgical protocol, of which intraoperative cultures were reported. We performed a meta-analysis for: (1) the rates of surprise positive culture nonunion, surprise infected nonunion, and contaminated culture nonunion, and (2) healing and (3) secondary surgery rates for each culture result. Risk of bias was assessed using the QUADAS-2 tool.

**Results:**

21 studies with 2,397 patients with a presumed aseptic nonunion were included. The rate of surprise positive culture nonunion was 16% (95%CI: 10–22%), of surprise infected nonunion 10% (95%CI: 5–16%), and of contaminated culture nonunion 3% (95%CI: 1–5%). The secondary surgery rate for surprise positive culture nonunion was 22% (95%CI: 9–38%), for surprise infected nonunion 14% (95%CI 6–22%), for contaminated culture nonunion 4% (95%CI: 0–19%), and for negative culture nonunion 6% (95CI: 1–13%). The final healing rate was 98% to 100% for all culture results. Coagulase-negative staphylococci accounted for 59% of cultured micro-organisms.

**Conclusion:**

These results suggest that surprise positive cultures play a role in the clinical course of a nonunion and that culturing is important in determining the etiology of nonunion, even if the pre-operative suspicion for infection is low. High healing rates can be achieved in presumed aseptic nonunions, regardless of the definitive intraoperative culture result.

## Introduction

Presence of infection is an important factor in the treatment of long-bone nonunions [1]. Infected nonunions are often managed with staged surgical treatment, whereas presumed aseptic nonunions are treated in a single stage [2, 3]. Infection can be confirmed pre- or intraoperatively in the presence of signs such as a fistula or sinus, wound breakdown, and purulent drainage or presence of pus [4]. Other factors considered are elevated serum inflammatory markers, radiological signs and suggestive local and systemic signs of infection such as erythema or fever. However, these factors are merely suggestive as they lack accuracy, specifically in detecting low-grade infections [5–7]. Consequently, if clinical signs of infection are absent, a nonunion is often presumed aseptic and in these cases, the definitive diagnosis of infection relies on intraoperative cultures from the nonunion site.

Several studies have reported on the rate and outcomes of nonunions that exhibit no clinical and/or laboratory signs of infection but reveal positive intraoperative cultures (referred to as “surprise” positive cultures) [8–10]. However, amongst these studies the definitions of presumed aseptic nonunions vary, as do local protocols for detection and treatment of positive cultures.

Therefore, our primary objective was to determine (1) the rate of “surprise” positive intraoperative cultures in presumed aseptic long-bone nonunion (surprise positive culture nonunion), and (2) the rate of “surprise” positive cultures that represent an infection (surprise infected nonunion) vs. a contamination (contaminated culture nonunion). Our secondary objectives were to determine the healing and secondary surgery rate for each culture result and to identify the cultured micro-organisms.

## Materials and methods

### Eligibility criteria

We included studies that reported on a (1) prospective or retrospective cohort of (2) ≥ 10 adult patients with a (3) presumed aseptic long-bone (clavicle, humerus, ulna, radius, femur or tibia) nonunion based on at least a clinical assessment, (4) treated with a single-stage surgical protocol, and (5) of which intraoperative cultures were reported. We excluded (1) review articles, (2) letters to the editor, (3) meeting abstracts, (4) technique papers, (5) studies not published in English, and (6) laboratory, cadaveric or animal studies.

### Information sources and search strategy

We searched MEDLINE (PubMed), Embase (OVID) and the Cochrane Database of Systematic Reviews and Central Register of Controlled Trials from 1980 until December 2021. The search syntax was based on terms including “nonunion”, “surgery”, and “infection” (Appendix 1). References of included studies were checked for publications missed by our search.

### Selection of studies

After duplicate removal, two reviewers (RW and CT) independently screened title and abstracts of the search results using the Rayyan web Application [11]. The same researchers independently assessed all full texts to confirm eligibility. Disagreements were resolved by consensus. If no consensus was reached, a third author (SJ) was consulted.

### Data collection

We extracted patient numbers for (1) presumed aseptic nonunions, (2) surprise positive culture nonunions, (3) surprise infected nonunions, (4) contaminated culture nonunions, and (5) negative culture nonunions. A surprise positive culture nonunion is defined as a presumed aseptic nonunion that reveals at least one surprise positive culture (regardless of representing an infection or contamination). A surprise infected nonunion is a presumed aseptic nonunion with a positive culture that represents an infection based on local study definitions (e.g., at least two cultures were positive) or has received treatment accordingly (e.g., long-term antibiotics). A contaminated culture nonunion is a presumed aseptic nonunion with a surprise positive culture that represents a contamination based on local study definitions and has therefore not received any treatment for infection.

For each culture result, we extracted the number of healed nonunions (at final follow-up) and nonunions requiring secondary surgeries (surgeries performed after the index procedure and before healing occurred). We collected numbers and types of cultured micro-organisms. We identified local protocols to differentiate between presumed septic and aseptic nonunion pre-operatively (with fracture-related infection [12] criteria as reference), and surprise infected nonunion and contaminated culture nonunion postoperatively. We also extracted culture and antimicrobial treatment strategies. Other data collected were: year of publication, study design, age and sex of included subjects, and anatomic region of the nonunions. Two reviewers (RK and CT) extracted data in Excel version 16.53 (Microsoft Corp., Redmond, WA, USA). Data extracted by one reviewer were checked by the other reviewer. Disagreement was resolved by consensus.

### Risk of bias assessment

Two reviewers (RK and CT) independently determined the risk of bias using the Quality Assessment of Diagnostic Accuracy Studies-2 (QUADAS-2 [13]). We modified the tool to fit the purpose of our study (Appendix 2), and used four domains: (1) patient selection, (2) index test (pre-operative assessment of infection), (3) reference standard (culture protocol), and (4) flow and timing. Two or more criteria were established for each domain. Each criterium was scored “yes”, “no”, or “unclear” and each domain was then scored as having a “high”, “low” or “unclear” risk of bias. No overall judgement of risk of bias was performed. Discrepancies were discussed until consensus was reached or by consulting a third reviewer (SJ).

### Synthesis methods

We performed a meta-analysis for: (1) rate of surprise positive culture nonunion, surprise infected nonunion and contaminated culture nonunion, and (2) healing and (3) secondary surgery rates for each culture result. Pooling of studies was performed in case ≥ 3 studies reported on the same outcome. An inverse variance, random effects model (DerSimonian and Laird method) was used for this purpose. This means that studies were weighted in inverse proportion to their variance to minimize the imprecision of the pooled effect estimate and that studies were allowed to have other factors (i.e. different populations, designs) contribute to the effect estimate [14]. To include effect estimates close to 0 or 100% the Freeman–Tukey double arcsine transformation was used [15]. The pooled effect estimates is presented as a percentage with a 95% confidence interval (CI). Stata version 17.0 (StataCorp., College Station, TX, USA) was used for meta-analyses and the accompanying forest plots and heterogeneity test (I2).

## Results

**Fig. 1:**
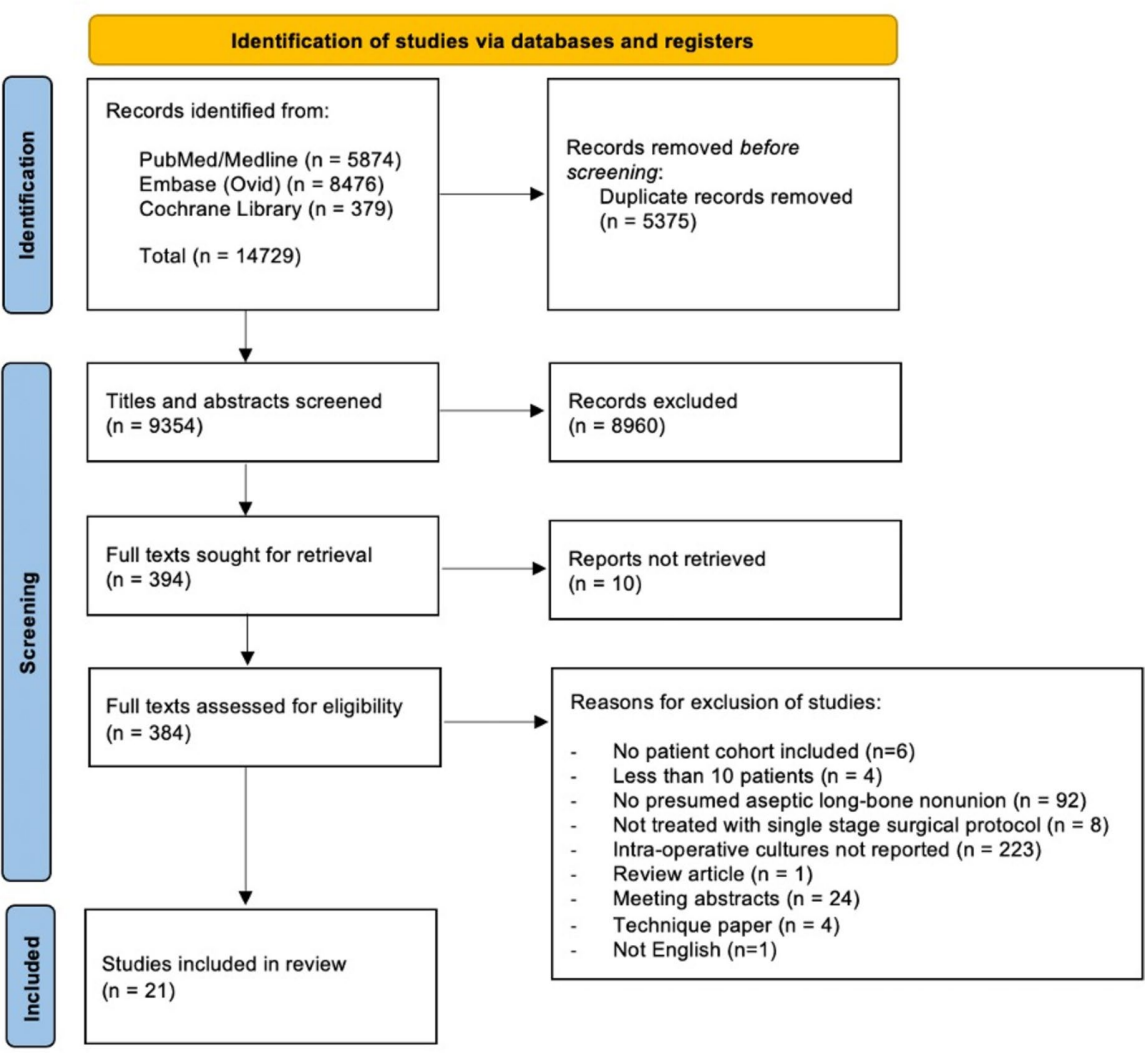
PRISMA Flowchart. Adapted from Page et al. [46]

Our search yielded 14,729 articles and after duplicate removal 9354 articles remained. The full texts of 384 studies were reviewed for inclusion. A total of 21 studies were included (Fig. 1).

### Study characteristics

The 21 studies included a total of 2397 patients (median: 49, range: 12–898) with a presumed aseptic nonunion (Table 1). Sex distribution was 62% males and 38% females based on 13 (61%) studies. Average ages ranged from 35 [16] to 50 [17] years. The most common anatomic location of presumed aseptic nonunions were the tibia (43%) and femur (40%).

**Table 1:** Individual study characteristics

Author	Year	Title	Design	Country	Total number of patients	Male (%)	Female (%)	Average age (range)	Tibia	Fibula	Tibia/fibula	Femur	Clavicle	Humerus	Radius/ulna	Other	only non-operative treatment (%)	infection history (%)	open fracture (%)
Amorosa et al. [9]	2013	A Single-Stage Treatment Protocol for Presumptive Aseptic Diaphyseal Nonunions: A Review of Outcomes	retrospective	United States	104	–	–	50 (14–96)	38	0	0	35	3	24	4	0	no	no	yes (25%)
Arsoy et al. [10]	2018	Outomes of Presumed Aseptic Long-Bone Nonunions With Positive Intraoperative Cultures Through a Single-Stage Surgical Protocol	retrospective	United States	898	–	–	–	–	–	–	–	–	–	–	–	–	no	yes (unknown %)
Bilgili et al. [24]	2020	Acute correction and intramedullary nailing of aseptic oligotrophic and atrophic tibial nonunions with deformity	retrospective	Turkey	17	12 (71)	5 (29)	36 (19–49)	17	0	0	0	0	0	0	0	no	no	yes (82%)
Fragomen et al. [25]	2019	The PRECICE magnetic IM compression nail for long bone nonunions: a preliminary report	retrospective	United States	14	9 (64)	5 (36)	49	5	0	0	9	0	0	0	0	no	yes (35%)	yes (43%)
Gille et al. [20]	2012	Is non-union of tibial shaft fractures due to nonculturable bacterial pathogens? A clinical investigation using PCR and culture techniques	retrospective	Germany	23	15 (65)	8 (35)	47 (20–82)	23	0	0	0	0	0	0	0	–	–	yes (36%)
Hackl et al. [26]	2021	The role of low-grade infection in the pathogenesis of apparently aseptic tibial shaft nonunion	retrospective	Germany	88	69 (78)	19 (22)	46 (18–81)	88	0	0	0	0	0	0	0	no	no	yes (42%)
Kim et al. [27]	2014	Indolent infection in nonunion of the distal femur	retrospective	Republic of Korea	22	12 (55)	10 (45)	44 (17–67)	0	0	0	22	0	0	0	0	no	no	yes (45%)
Mills et al. [18]	2016	The multifactorial aetiology of fracture nonunion and the importance of searching for latent infection	retrospective	United Kingdom	75^a^	–	–	–	–	–	–	–	–	–	–	–	yes (unknown %)	yes (11%)	yes (unknown %)
Moghaddam et al. [29]	2017	Treatment of atrophic femoral non-unions according to the diamond concept: Results of one- and two-step surgical procedure	retrospective	Germany	41^b^	22 (54)	19 (46)	44 (19–76)	0	0	0	41	0	0	0	0	no	–	yes (20%)
Moghaddam et al. [3]	2015	Treatment of atrophic tibia non-unions according to 'diamond concept': Results of one- and two-step treatment	retrospective	Germany	49^b^	32 (65)	17 (35)	46 (15–76)	49	0	0	0	0	0	0	0	yes (2%)	–	yes (33%)
Hierholzer et al. [19]	2006	Plate Fixation of Ununited Humeral Shaft Fractures: Effect of Type of Bone Graft on Healing	retrospective	United States	78	27 (35)	51 (65)	–	0	0	0	0	0	78	0	0	yes (51%)	yes (5%)	yes (unknown %)
Morgenstern et al. [21]	2018	The value of quantitative histology in the diagnosis of fracture-related infection	unclear	United Kingdom	114^c^	–	–	–	–	–	–	–	–	–	–	–	–	yes (unknown %)	yes (unknown %)
Mittal et al. [28]	2021	Management of Refractory Aseptic Subtrochanteric Non-union by Dual Plating	retrospective	India	12	6 (50)	6 (50)	43 (18–65)	0	0	0	12	0	0	0	0	no	–	–
Olszewski et al. [8]	2016	Fate of Patients With a "Surprise" Positive Culture After Nonunion Surgery	retrospective	United States	453	–	–	–	–	–	–	–	–	–	–	–	yes (unknown %)	yes (unknown %)	yes (unknown %)
Otchwemah et al. [30]	2020	High prevalence of bacteria in clinically aseptic non-unions of the tibia and the femur in tissue biopsies	retrospective	Germany	18	–	–	44	11	0	0	7	0	0	0	0	no	–	yes (50%)
Schulz et al. [32]	2009	Is the Wave Plate Still a Salvage Procedure for Femoral Non-union? Results of 75 Cases Treated with a Locked Wave Plate	retrospective	Germany	75	57 (76)	18 (24)	44 (17–81)	0	0	0	75	0	0	0	0	no	–	yes (35%)
Tanner et al. [22]	2021	The Influence of an Occult Infection on the Outcome of Autologous Bone Grafting During Surgical Bone Reconstruction: A Large Single-Center Case–Control Study	retrospective	Germany	109^b^	–	–	–	–	–	–	–	–	–	–	–	–	no	yes (unknown %)
Shin et al. [31]	2021	Is open bone graft always necessary when treating aseptic subtrochanteric nonunion with a reamed intramedullary nail?	retrospective	Republic of Korea	37	22 (59)	15 (41)	–	0	0	0	37	0	0	0	0	no	no	no
Tosounidis et al. [23]	2021	Can CRP Levels Predict Infection in Presumptive Aseptic Long Bone Non-Unions? A Prospective Cohort Study	prospective	United Kingdom	105	59 (56)	46 (44)	47 (16–92)	37	0	0	56	0	0	0	12	–	no	–
Wenter et al. [17]	2016	[18F] FDG PET accurately differentiates infected and non-infected non-unions after fracture fixation	retrospective	Germany	25^d^	–	–	50	17	4	2	7	0	1	0	4	–	yes (25%)	–
Zelle et al. [16]	2003	Exchange Reamed Nailing for Aseptic Nonunion of the Tibia	retrospective	United States	40	30 (75)	10 (25)	35.1	40	0	0	0	0	0	0	0	no	no	yes (68%)
**Total**	**–**	**–**	**–**	**–**	**2397**	**62%**	**38%**	**–**	**325**	4	**2**	**301**	**3**	**103**	**4**	**16**	**–**	**–**	**–**

^a^we included all patients without active ongoing infection. This included patients with previous treatment for infection who were considered to be free of infection

^b^we only included patients that received single stage treatment

^c^we only included patients of which infection was not confirmed by clinical appearance

^d^we only included patients of which tissue cultures were taken

–: missing / not reported

Four (19%) studies [3, 8, 18, 19] included patients that had not been treated surgically prior to the index procedure. Six (29%) studies [10, 17, 20–23] did not provide this information. In the remaining 11 studies [9, 16, 24–32] patients had undergone at least one prior surgical procedure.

Six (29%) studies [8, 17–19, 21, 25] included patients with a history of infection (range of patients with prior infection: 5% [19] to 36% [25]). Six (29%) studies [3, 20, 28–30, 32] did not report on infection history. In the nine remaining studies patients with history of infection were not included [9, 10, 16, 22–24, 26, 27, 31].

Seventeen (81%) studies [3, 8–10, 16, 18–22, 24–27, 29, 30, 32] included patients with initially open fractures (range of patients with open fracture: 20% [29] to 82% [24]). Three  studies [17, 23, 28] did not report this and one study [31] only included patients with closed fractures.

### Risk of bias

**Fig. 2:**
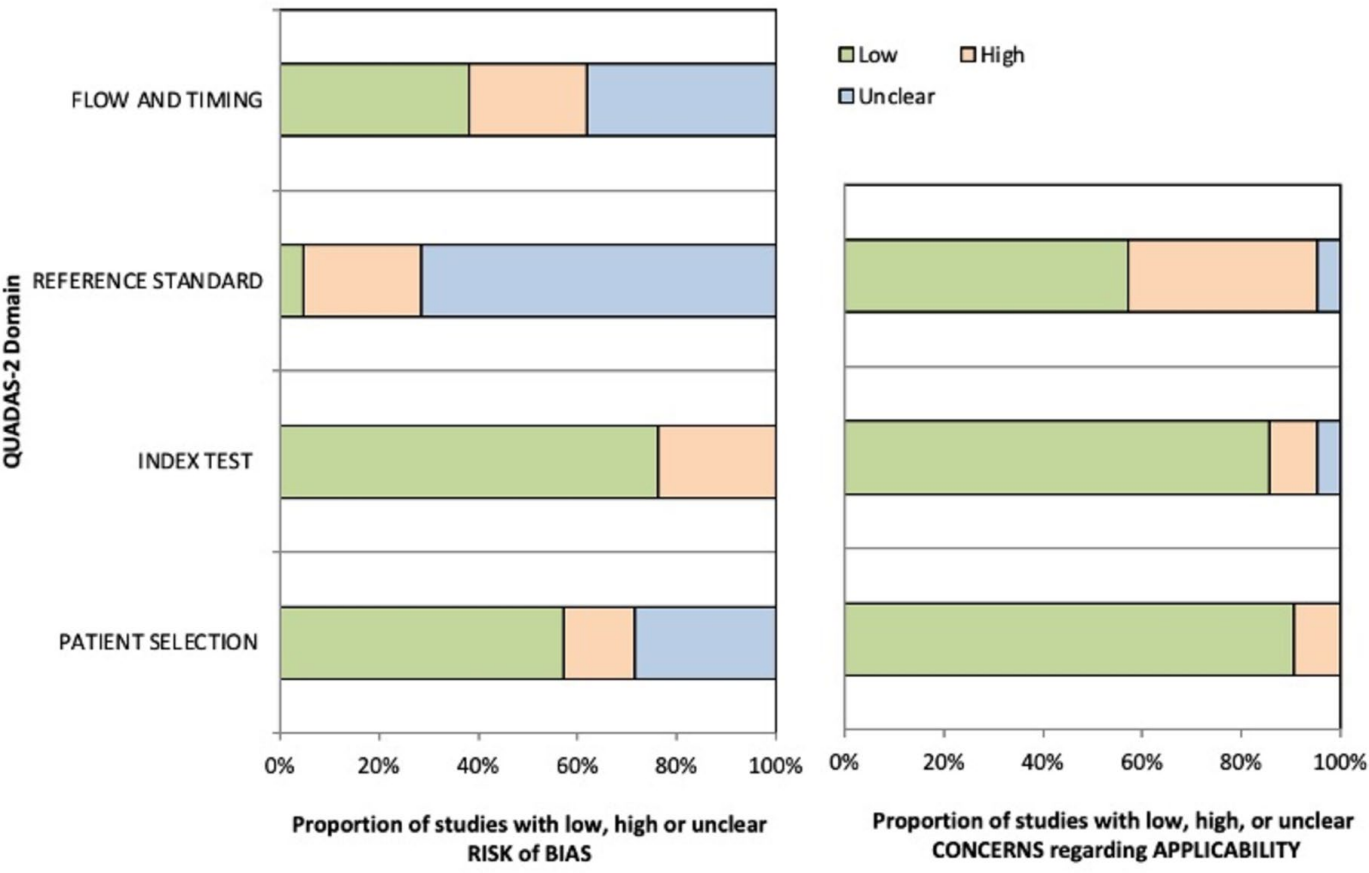
Risk of bias and concerns regarding applicability across studies. Figure adapted from Whiting et al. [13]. Flow and timing: inclusion of patients in methods and analysis. Index test: conduct and interpretation of the pre-operative assessment for infection. Reference standard: conduct of the culture protocol. Patient selection: patient selection and inclusion criteria. Applicability: the reference standard, the index test, or patient selection should match the review question. See appendix 2 for a full description of the criteria for each domain

For each of the four domains, less than 25% of studies had a high risk of bias. The lowest risk of bias was found for the index test (pre-operative assessment of infection) with 75% of studies having a low risk of bias (Fig. 2 and appendix 3). Although the clinical assessment generally lacked a detailed description, for most studies it was clear if infection was ruled out based on suggestive (e.g., laboratory values) and confirmatory signs, or only on confirmatory signs. An unclear risk of bias was found for the reference standard (cultures from the nonunion site), with 71% of studies having an unclear risk of bias. In general, there was a low concern that studies were not applicable for patient selection and the index test. The concern for applicability was high in 48% of studies for the reference standard.

### Outcomes

#### Rate of surprise positive cultures, surprise infected nonunions and contaminated cultures

**Fig. 3:**
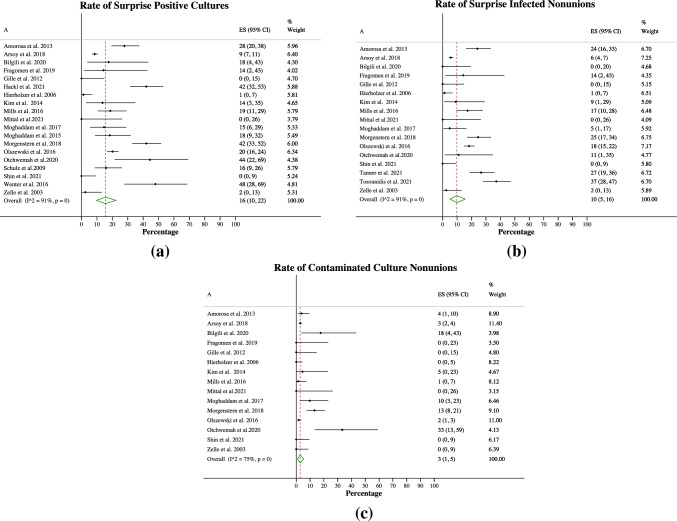
Rates of surprise positive culture nonunion, surprise infected nonunion and contaminated culture nonunion

The rate of surprise positive cultures was 16% (10–22%, Fig. 3a, 19 studies and 2183 patients) (Table 2). The rate of surprise infected nonunions was 10% (5–16% Fig. 3b, 17 studies and 2160 patients). The rate of contaminated culture nonunions was 3% (1–5% Fig. 3c, 15 studies and 1964 patients). Note that due to underlying data the cumulative percentage of surprise infected nonunions and contaminated culture nonunions is not the same as the total number of surprise positive culture nonunions (i.e., some studies did not differentiate between infection vs. contamination whereas others only provided the rate of surprise infected nonunions, see Table 2).

**Table 2:** Individual study results

Study		Culture results			Final union rate				Secondary surgery rates for persistent nonunion			
Author	Year	Culture positive nonunion	Infected nonunion	Contaminated culture nonunion	Culture positive nonunion	Infected nonunion	Contaminated culture nonunion	Culture negative nonunion	Culture positive nonunion	Infected nonunion	Contaminated culture nonunion	Culture negative nonunion
Amorosa et al. [9]	2013	28%	24%	4%	100%	100%	100%	–	28%	33%	0%	6%
Arsoy et al. [10]	2018	9%	6%	3%	99%	100%	96%	–	18%	22%	12%	–
Bilgili et al. [24]	2020	18%	0%	18%	100%	–	100%	100%	33%	–	33%	21%
Fragomen et al. [25]	2019	14%	14%	0%	100%	100%	–	92%	0%	0%	–	17%
Gille et al. [20]	2012	0%	0%	0%	–	–	–	–	–	–	–	–
Hackl et al. [26]	2021	42%	–	–	97%	–	–	100%	57%	–	–	29%
Kim et al. [27]	2014	14%	9%	5%	100%	100%	100%	100%	33%	50%	0%	0%
Mills et al. [18]	2016	19%	17%	1%	–	–	–	–	–	–	–	–
Moghaddam et al. [29]	2017	15%	5%	10%	100%	100%	100%	94%	–	–	–	–
Moghaddam et al. [3]	2015	18%	–	–	56%	–	–	90%	–	–	–	–
Hierholzer et al. [19]	2006	1%	1%	0%	100%	100%	–	99%	0%	0%	–	1%
Morgenstern et al. [21]	2018	42%	25%	13%	–	–	–	–	–	–	–	–
Mittal et al. [28]	2021	0%	0%	0%	–	–	–	100%	–	–	–	0%
Olszewski et al. [8]	2016	20%	18%	2%	92%	95%	63%	100%	22%	20%	–	4%
Otchwemah et al. [30]	2020	44%	11%	33%	–	–	–	–	–	–	–	–
Schulz et al. [32]	2009	16%	–	–	100%	–	–	100%	–	–	–	–
Tanner et al. [22]	2021	–	27%	–	–	86%	–	–	–	–	–	–
Shin et al. [31]	2021	0%	0%	0%	–	–	–	89%	–	–	–	–
Tosounidis et al. [23]	2021	–	37%	–	–	100%	–	–	–	–	–	–
Wenter et al. [17]	2016	48%	–	–	–	–	–	–	–	–	–	–
Zelle et al. [16]	2003	3%	3%	0%	100%	100%	–	95%	0%	0%	–	3%

For studies that did not provide an explicit differentiation between contaminated cultures and infected nonunions we considered cases with a surprise positive culture that were treated for infection as infected (e.g., long-term antibiotics)

–: missing / not reported

#### Secondary surgery rate for persistent nonunion

**Fig. 4:**
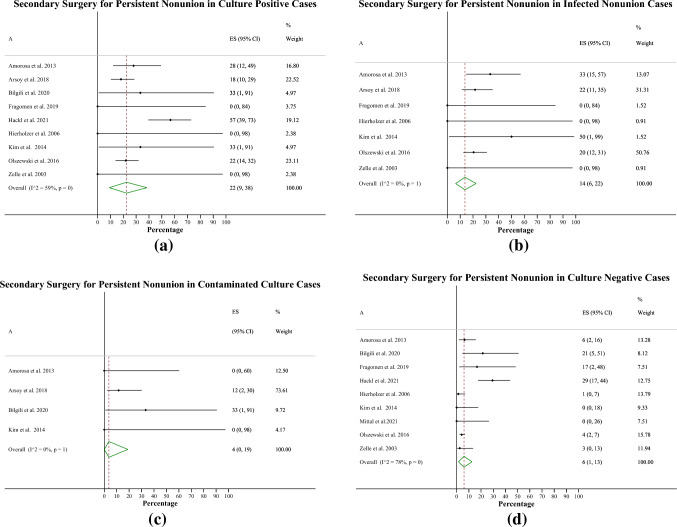
Secondary surgery rates

The rate of secondary surgery for nonunions with a surprise positive culture was 22% (9–38%, Fig. 4a, 9 studies and 240 patients), for surprise infected nonunions 14% (6–22%, Fig. 4b, 7 studies and 161 patients), for contaminated culture nonunions 4% (0–19% Fig. 4c, 4 studies and 34 patients), and for negative culture nonunions 6% (1–13%, Fig. 4d, 9 studies and 648 patients).

#### Final healing rate

**Fig. 5:**
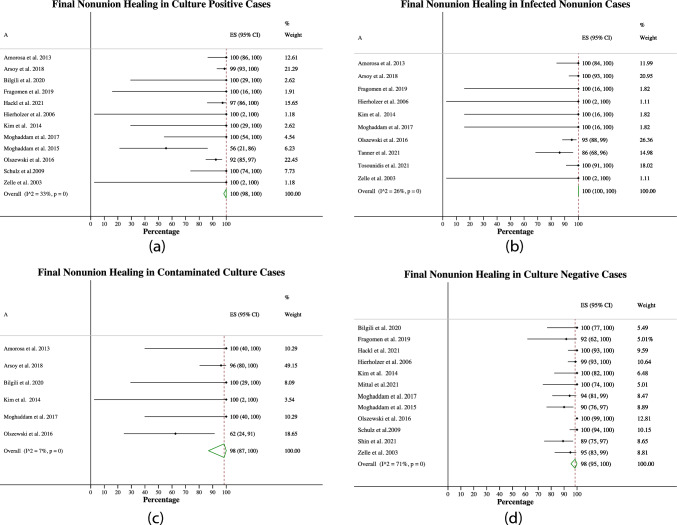
Final healing rates

For nonunions with a surprise positive culture, the final healing rate was 100% (98–100%, Fig. 5a), based on 12 studies and 267 patients. For surprise infected nonunions, this was 100% (100–100%, Fig. 5b), based on 10 studies and 231 patients. For contaminated culture nonunions, this was 98% (87–100%, Fig. 5c), based on six studies and 46 patients. For negative culture nonunions, this was 98% (95–100%, Fig. 5d), based on 12 studies and 761 patients.

#### Cultured micro-organisms

**Fig. 6:**
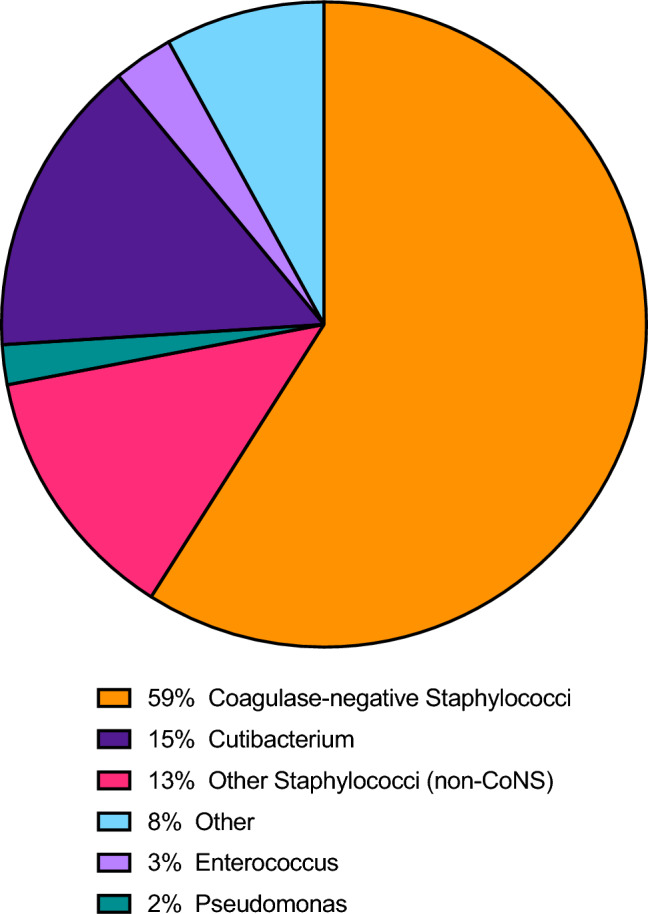
Pie-chart of the cultured micro-organisms displayed per genus. Includes micro-organisms described as Propionibacterium without further specification, assuming that unspecified Propionibacterium would consist mostly of cutaneous species, that are now classified as Cutibacterium [47]

We did not differentiate the identified micro-organisms between those found in definitive “surprise” infected nonunions and in contaminated cultures. Fifteen (71%) studies described the numbers of micro-organisms that were cultured [3, 8–10, 16, 17, 19, 21, 23, 24, 26, 27, 29, 30, 32]. The most common genus cultured was Staphylococcus (72%), followed by Cutibacterium (15%) (Table 3 and Fig. 6). Coagulase-negative staphylococci (CoNS) accounted for 59% of all cultured micro-organisms.

**Table 3:** Cultured micro-organisms

Species (in case not specified, the genus is displayed)	% of surprise positive culture nonunions with the micro-organism
Coagulase-negative staphylococci (unspecified)	38.1%
Staphylococcus epidermidis	12.7%
Methicillin-sensitive Staphylococcus aureus	10.1%
Cutibacterium acnes^a^	9.3%
Cutibacterium (unspecified)^b^	6.0%
Staphylococcus capitis	4.2%
Enterococcus (faecalis & faecum combined)	2.8%
Methicillin-resistant Staphylococcus aureus	2.6%
Pseudomonas (unspecified)	2.3%
Bacillus	1.5%
Staphylococcus (unspecified)	1.0%
Staphylococcus hominis	1.0%
Peptostreptococcus (unspecified)	1.0%
Streptococcus viridans	0.9%
Clostridium (unspecified)	0.7%
Enterobacter cloacae	0.7%
Streptococcus agalactiae	0.6%
Staphylococcus haemolyticus	0.8%
Staphylococcus lugdunensis	0.5%
Candida (unspecified)	0.3%
Fungi: Aspergillus (unspecified)	0.3%
Escherichia coli	0.3%
Staphylococcus simulans	0.3%
Staphylococcus oralis	0.3%
Staphylococcus cohnii	0.3%
Staphylococcus caprae	0.3%
Serratia (unspecified)	0.3%
Prevotella buccae	0.3%
Peptostreptococcus prevotii	0.2%
Total	100%

^a^Includes micro-organisms described as the formerly known Propionibacterium acnes[47]

^b^Includes micro-organisms described as Propionibacterium without further specification, assuming that unspecified Propionibacterium would consist mostly of cutaneous species, that are now classified as Cutibacterium[47]

#### Differentiation between presumed septic and aseptic nonunion pre-operatively

Eight (38%) studies [3, 9, 10, 17, 21, 27, 29, 30] described explicit clinical criteria in the pre-operative assessment of infection (Table 4). Of these, Morgenstern et al. [21] only used confirmatory clinical criteria rule out infection. The remaining seven studies (also) used suggestive clinical criteria (i.e., erythema, fever, hyperthermia). Of these seven studies, four studies [3, 27, 29, 30] described only suggestive criteria, however it is to assume that in these studies patients with confirmatory clinical signs of infection would not have been presumed aseptic.

**Table 4:** Differentiation between presumed septic and aseptic nonunion pre-operatively

			Confirmatory criteria			Suggestive criteria							
Author	Year	Description of clinical assessment	Fistula/sinus	Wound breakdown	Purulent drainage / presence of pus	Pain	Erythema	Swelling	Local hyperthermia	Fever	Non-purulent drainage	Radiological signs	Laboratory values
Amorosa et al. [9]	2013	Surrounding erythema over the scar, fluctuence, drainage or sinus	yes	–	yes	–	yes	yes	–	–	–	–	–
Arsoy et al. [10]	2018	Fever, draining wound, and sinus	yes	–	yes	–	–	–	–	–	yes	–	–
Bilgili et al. [24]	2020	Clinical evidence for infection										–	yes
Fragomen et al. [25]	2019	Active infection										–	yes
Gille et al. [20]	2012	Clinical signs of infection										–	–
Hackl et al. [26]	2021	Clinical evidence for local infection										–	–
Kim et al. [27]	2014	Physical signs of erythema or local heat around the scar	–	–	–	–	yes	-	yes	–	–	–	yes
Mills et al. [18]	2016	Clinical suspicion for infection										–	yes
Moghaddam et al. [29]	2017	Clinical signs of infection (warmth, swelling and redness)	–	–	–	–	yes	yes	yes	–	–	–	–
Moghaddam et al. [3]	2015	Clinical signs of infection (warmth, swelling and redness)	–	–	–	–	yes	yes	yes	–	–	–	–
Hierholzer et al. [19]	2006	Clinical evidence of infection										yes	yes
Morgenstern et al. [21]	2018	Fracture related infection confirmatory criteria: fistula, sinus, wound breakdown, purulent drainage or the presence of pus	yes	yes	yes	–	–	–	–	–	–	–	–
Mittal et al. [28]	2021	Clinical examination										yes	yes
Olszewski et al. [8]	2016	Clinical evidence for infection											yes
Otchwemah et al. [30]	2020	Local hyperaemia, warmth, swelling, and pain on palpation	–	–	–	yes	yes	yes	yes	–	–	–	–
Schulz et al. [32]	2009	Clinical signs of infection										–	–
Tanner et al. [22]	2021	Soft tissue, mechanical stability and function										–	yes
Shin et al. [31]	2021	Clinical examination										–	yes
Tosounidis et al. [23]	2021	Local and/or systemic signs of infection										–	–
Wenter et al. [17]	2016	Local fistulas or pyrophoric wounds, erythema, and/or hyperthermia	yes	–	–	–	yes	–	yes	–	–	–	–
Zelle et al. [16]	2003	Clinical markers										–	yes

Variables are based on recently published fracture-related infection (FRI) consensus criteria

Names of individual FRI criteria are shortened for presentation purposes

Yes: explicitly described in assessment

–: not described in assessment

Blank: no detailed description of clinical criteria provided

The 13 (62%) remaining studies did not explicitly describe the clinical criteria used in the assessment (i.e., presumed aseptic nonunion defined by the absence of “clinical signs of infection” or “active infection”). Ten (48%) studies [8, 16, 18, 19, 22, 24, 25, 27, 28, 31] described the use of laboratory values and two (10%) studies [19, 28] the use of radiological findings to rule out infection pre-operatively.

#### Local culture strategy

Eleven (52%) of studies provided information on culture protocols (Table 5). Four (19%) studies [9, 10, 22, 23] reported taking at least five cultures. Five (24%) studies [18, 20, 22, 26, 30] described culturing for at least 14 days. In two studies [26, 27] antibiotics were administered before cultures were taken. One study used sonication as a separate culture result [21].

**Table 5:** Local culture protocols, and differentiation and treatment for surprise infected nonunions

		Culture protocol			Differention between infection and contamination			Treatment
Author	Year	Antibiotics administered before culture samples are taken?	Number of culture samples taken during surgery	Minimal duration of culturing (days)	Definition of contamination vs infection provided	Consultation with infectious disease specialist	Differentiation based on number of positive cultures	Treatment for surprise infected nonunions without clinical signs of infection
Amorosa et al. [9]	2013	no	5	–	yes	yes	yes ≥ 2 = infected	Antibiotics
Arsoy et al. [10]	2018	no	≥ 5	–	yes	yes	yes 2 = contaminated or infected 3 = infected	Antibiotics
Bilgili et al. [24]	2020	–	–	–	no	–	–	–
Fragomen et al. [25]	2019	–	–	–	no	–	–	Antibiotics
Gille et al. [20]	2012	no	≥ 3	14	no	–	–	–
Hackl et al. [26]	2021	yes	4	≥ 14	yes	no	yes, ≥ 2 = infected	Antibiotics
Kim et al. [27]	2014	yes	3–5	–	yes	yes	yes, ≥ 2 = infected	Antibiotics
Mills et al. [18]	2016	no	≥ 3	14	yes	no	yes, ≥ 2 = infected	–
Moghaddam et al. [29]	2017	–	–	–	no	–	–	Antibiotics
Moghaddam et al. [3]	2015	–	–	–	no	–	–	–
Hierholzer et al. [19]	2006	–	–	–	no	–	–	Antibiotics
Morgenstern et al. [21]	2018	no	3–5^a^	–	yes	no	yes, ≥ 2 = infected	–
Mittal et al. [28]	2021	–	–	–	no	–	–	–
Olszewski et al. [8]	2016	–	≥ 3	≥ 5	yes	yes	no	Antibiotics
Otchwemah et al. [30]	2020	no	multiple	14	yes	no	yes ≥ 2 = infected	–
Schulz et al. [32]	2009	–	–	–	no	–	–	–
Tanner et al. [22]	2021	no	≥ 5	14	yes	yes	yes ≥ 2 = infected	–
Shin et al. [31]	2021	no	–	–	no	–	–	–
Tosounidis et al. [23]	2021	no	≥ 5	–	yes	yes	unclear	Antibiotics
Wenter et al. [17]	2016	–	–	–	no	–	–	–
Zelle et al. [16]	2003	–	–	–	no	–	–	Antibiotics

^a^sonication results were considered a separate culture result

Blank: missing/not reported

#### Differentiation between infection and contamination

Ten studies (48%) explicitly provided a differentiating definition for infection and contamination (Table 5), of which eight studies [9, 10, 18, 21, 22, 26, 27, 30]  required at least two cultures to be positive to deem a nonunion as infected and six studies [8–10, 22, 23, 27] described consultation with an infectious disease specialist.

#### Antimicrobial therapy

None of the studies reported on the use of empirical antibiotics beyond the perioperative period whilst awaiting culture results. Ten (48%) studies [8–10, 16, 19, 23, 25–27, 29] reported treating patients with a surprise infection primarily with antibiotics, if clinical signs of infection remained absent. This information was not provided by the remaining studies (Table 5).

## Discussion

The surgical treatment protocol for a long-bone nonunion largely depends on the absence or presence of infection. If confirmatory clinical signs of infection are absent, a nonunion is often presumed aseptic. We established that, in these cases, surprise positive cultures occur in approximately 1 in 6, and surprise infected nonunions in 1 in 10 patients. We found that 1 in 5 patients with a surprise positive culture nonunion and 1 in 7 patients with a surprise infected nonunion required secondary revision surgery, compared to 1 in 17 patients with a negative culture nonunion. It may be possible that revision surgery was performed only because a positive culture was found. However, studies primarily initiated antibiotic treatment specific to the identified micro-organisms in case of a surprise infected nonunion that remained without clinical signs of infection. The need for additional surgery may be explained by the fact that none of the studies reported an empiric antibiotic treatment protocol until cultures return. Staphylococci – which were the most cultured micro-organisms – are able to develop a biofilm within days, which can only be eradicated by removal or exchange of implants and biofilm active antibiotic therapy (e.g., rifampicin) [35, 36]. In addition, vascularity of nonunions may be compromised, which limits local penetration of systemic antibiotics. Consequently, initiating antibiotic treatment only after cultures return might be beyond the “window of opportunity”. For confirmed FRIs, it is therefore recommended that surgical debridement should be followed by empiric broad spectrum intravenous antimicrobial therapy [37, 38]. In this systematic review, we found that final healing rates are close to 100% for presumed aseptic nonunions regardless of the culture result, and despite none of the studies reporting empiric antibiotic treatment. This would suggest that the combination of antibiotics tailored to the micro-organism(s) found with additional revision surgery is effective and that empiric broad-spectrum antibiotics may not be necessary. This is important in the context of antibiotic stewardship, cost-reduction, and prevention of side-effects. Further studies have to assess the local epidemiology, antibiotic resistance rates, and patient characteristics of surprise infected nonunions to establish tailored protocols [38].

We aimed to compare the criteria to define a presumed aseptic nonunion against the FRI criteria [4]. However, over 60% of studies did not specify these criteria. This is problematic as these descriptions (e.g., “clinical signs of infection” or “active infection”) may reflect both confirmatory or suggestive clinical signs of infection and therefore do not represent a repeatable threshold. Of the studies that did specify the criteria, most excluded patients based on suggestive clinical signs for infection. In general, a limitation of the suggestive FRI criteria is that some criteria (e.g., pain, swelling, redness, bone lysis around implants) may also be a result of the nonunion itself. Half of studies used laboratory values to rule out infection pre-operatively. The accuracy of these diagnostic test remains debatable. The study of Tosounidis et al. found 26% surprise infected nonunions, even if CRP was normal [23]. Hackl et al. found no significant differences in laboratory values between surprise infected nonunion and negative culture nonunions [26]. Others have also confirmed that laboratory values are not accurate to diagnose low-grade infection [5]. These findings show that adequate pe-operative diagnosis of infection remains difficult if confirmatory signs of infection are absent. Nonunion may even be the only symptom of the infection in these cases.

Given these findings, the definitive diagnosis of infection still heavily relies on intraoperative cultures. We found that only half of the studies reported a culture strategy protocol, and these protocols often lacked detail. Consequently, the protocols did not meet current recommendations [1, 4, 39]. Inappropriate sampling may underestimate (e.g., inadequate or insufficient samples, or short culture duration) the rate of surprise infected nonunions. It is recommended to take at least five cultures from the bone-implant interface directly after the incision and incubate these for 10–14 days [4]. Only four studies reported taking five or more cultures and only five studies reported long-term culturing. Long-term culturing is important to detect slow growing micro-organisms, such as Cutibacterium species and Coagulase-negative staphylococci [40–42]. Consequently, the prevalence of slow growing micro-organisms might be underestimated. Nevertheless, we found that over half of the identified micro-organisms were low virulent Coagulase-negative staphylococci [43]. Indeed, late (inherent to a nonunion) infections are most often caused by a low virulent micro-organism [35, 39]. Interestingly, 12% of infections were still caused by virulent *Staphylococcus aureus*. These micro-organisms may cause a low-grade infection when a low inoculum is introduced during the initial trauma or earlier surgery [35]. Although from the data of the present study it cannot be determined if a surprise infection truly causes nonunion, our findings do suggest that in order to adequately determine the etiology of a nonunion, prolonged culturing is necessary.

After cultures return positive, it is important to differentiate between contamination and infection as this has consequences for the treatment strategy. Only half of the studies explicitly reported criteria for such a differentiation. Most of these studies required two or more cultures to be positive to deem a nonunion as infected. This is in line with recent recommendations [4]. This is justifiable when comparing the secondary surgery rate for contaminated culture nonunions (4%, 95%CI 0–19%) and negative culture nonunions (6%, 95%CI: 1–13%).

Our study has several limitations. First, the major limitation of this study is that – although presented as single population – presumed aseptic nonunions are naturally a heterogenous group with varying characteristics; which is inherently demonstrated by our results. Accordingly, the included studies varied in patient history (e.g., infection or surgical history, open fractures), in definition of presumed aseptic nonunion, and in culture protocols. These differences may explain the substantial statistical heterogeneity we found in our meta-analysis of pooled rate [14]. Clinicians should therefore interpret these results in light of their own definitions for a presumed aseptic nonunion. Second, we excluded many studies because the authors did not report on intraoperative culture results. Reporting on intraoperative cultures may have been omitted if no positive cultures are found, which is considered publication bias. Similarly, publication bias may lead to an overestimation of the union rate as authors tend to publish successful treatment results. Third, we included studies that were published within a large timeframe (e.g., we also included studies published twenty years ago). Since then, treatment and diagnostic strategies have evolved and this may influence individual study results. Fourth, the majority of presumed aseptic nonunions affected the lower extremity. Upper extremity FRIs are often caused by different organisms (e.g., *Cutibacterium acnes* [44, 45]) and this limitation should be considered when extrapolating results to other anatomic regions. Last, we were unable to stratify the cultured micro-organisms into causative versus contaminant as this was not consistently reported by the individual studies.

## Conclusion

We found that in presumed aseptic nonunion cases, surprise positive intraoperative cultures occur in approximately 1 in 6 patients and surprise infections in 1 in 10 patients. The cultured organisms are most often of low virulence and Coagulase-negative staphylococci account for 59% of all cultured micro-organisms. Patients with a surprise positive culture and surprise infection require secondary surgeries more often compared to patients with a negative culture nonunion, although final healing rates are comparably high. Combined, these findings suggest that surprise positive cultures play a role in the clinical course of a nonunion, that (long-term) culturing is important in determining the etiology of nonunion even if the pre-operative suspicion for infection is low, and that eventually high healing rates can be achieved in presumed aseptic nonunions, regardless of the definitive intraoperative culture result.

### Other information

#### Protocol and registration

We use the Preferred Reporting Items for Systematic Reviews and Meta-Analysis (PRISMA) guideline for designing and reporting systematic reviews [46]. We registered our protocol on PROSPERO (registration number: CRD42021251319) prior to study selection. We made one protocol deviation as we performed our meta-analysis with STATA and included a Freeman–Tukey double arcsine transformation to include studies in which proportions are equal to 0 or 100% [15].

## Data Availability

The data that support the findings of this study are available from the corresponding author upon reasonable request.
